# REFRAMING AGING AND VISION LOSS AS A PUBLIC HEALTH IMPERATIVE

**DOI:** 10.1093/geroni/igad104.2926

**Published:** 2023-12-21

**Authors:** Priscilla Rogers

**Affiliations:** VisionServe Alliance, Mooresburg, Tennessee, United States

## Abstract

Severe vision impairment and blindness often have profound effects upon older people and those who care for and about them. Recent translational research reveals in the United States, 7.3% of older people report severe vision impairment or blindness. A recent study estimated that the population of people with vision impairment will increase by 118% by 2050. The greatest increases will be among the most senior, women, African Americans, and Hispanics. People with vision impairment report greater prevalence of chronic conditions, poorer health, and poorer quality of life than older people without vision impairment, and they are much more likely to experience multiple disabilities. These factors point to grave disparities in health equity. While the multiple studies have informed social, health, and economic disparities among older people with vision impairment at the national level, virtually nothing is known about the variability of the prevalence of vision impairment, chronic conditions, health-related quality of life, and disability at the state level. The geographic distribution of vision impairment varies among and within states. Often rural and poorer areas have a higher prevalence of vision impairment — areas that generally have a paucity of eye care providers, social support systems, and vision rehabilitation services. As aging issues are addressed at state and federal levels, vision loss and vision rehabilitation are frequently omitted from the conversations and plans (e.g., Master Plans on Aging and/or Age-Friendly Plans) and from policy instruments such as the Older Americans Act, which has no mention of vision or vision loss.

